# Attaining 95-95-95 through Implementation Science: 15 Years of Insights and Best Practices from the Walter Reed Army Institute of Research’s Implementation of the U.S. President’s Emergency Plan for AIDS Relief

**DOI:** 10.4269/ajtmh.20-0541

**Published:** 2020-11-09

**Authors:** Elizabeth H. Lee, Kavitha Ganesan, Samoel A. Khamadi, Stanley C. Meribe, Dorothy Njeru, Yakubu Adamu, Fred Magala, Trevor A. Crowell, Eniko Akom, Patricia Agaba, Priyanka Desai, Tiffany Hamm, Deydre Teyhen, Julie A. Ake, Christina S. Polyak, Douglas Shaffer, Fredrick Sawe, Patrick W. Hickey

**Affiliations:** 1 U.S. Military HIV Research Program, Walter Reed Army Institute of Research, Silver Spring, Maryland;; 2 The Uniformed Services University of the Health Sciences, Bethesda, Maryland;; 3 Henry M. Jackson Foundation for the Advancement of Military Medicine, Bethesda, Maryland;; 4 HJF Medical Research International, Mbeya, Tanzania;; 5 U.S. Army Medical Research Directorate-Africa, Abuja, Nigeria;; 6 U.S. Army Medical Research Directorate-Africa, Nairobi, Kenya;; 7 Makerere University Walter Reed Project, Kampala, Uganda;; 8 U.S. Department of Health and Human Services, Nairobi, Kenya;; 9 HJF Medical Research International, Kericho, Kenya

## Abstract

The Walter Reed Army Institute of Research (WRAIR) supports more than 350,000 people on lifesaving HIV treatment in Kenya, Nigeria, Tanzania, and Uganda through funding from the U.S. President’s Emergency Plan for AIDS Relief (PEPFAR). Here, we review and synthesize the range of impacts WRAIR’s implementation science portfolio has had on PEPFAR service delivery for military and civilian populations since 2003. We also explore how investments in implementation science create institutional synergies within the U.S. Department of Defense, contributing to broad global health engagements and improving health outcomes for populations served. Finally, we discuss WRAIR’s contributions to PEPFAR priorities through use of data to drive and improve programming in real time in the era of HIV epidemic control and public health messaging that includes prevention, the 95-95-95 goals, and comorbidities.

## INTRODUCTION

Since HIV/AIDS was first recognized, more than 75 million people have been infected, and 32 million have died worldwide.^1^ The Joint UN Programme on HIV/AIDS (UNAIDS) estimates that the number of people living with HIV (PLHIV) has increased from 7.9 million in 1990 to 37.9 million in 2018. Concurrently, annual new infections have declined from a peak of 2.9 million in the late 1990s to 1.7 million in 2018, and HIV-associated deaths have declined over 55% from a peak of 1.7 million in 2003–2004.^2^

As the United States’ largest foreign aid program, the U.S. President’s Emergency Plan for AIDS Relief (PEPFAR) uses a whole-of-government approach and has become a model for foreign aid programs through focus on person-centered health impact, fiscal efficiency, and accountability.^3^ Since 2003, PEPFAR’s leadership in the global HIV response has transformed the previously unchecked pandemic so that control is now in sight. Countries are achieving or nearing the UNAIDS “90-90-90 by 2020” goals: 90% of PLHIV will know their status, 90% of those who know their status will be on treatment, and 90% of those on treatment will be virally suppressed.^4^ These achievements necessitated an unprecedented implementation science program to improve scale, pace, and efficiency to achieve even more ambitious goals of 95-95-95 by 2030.^5^

**Figure 1.**
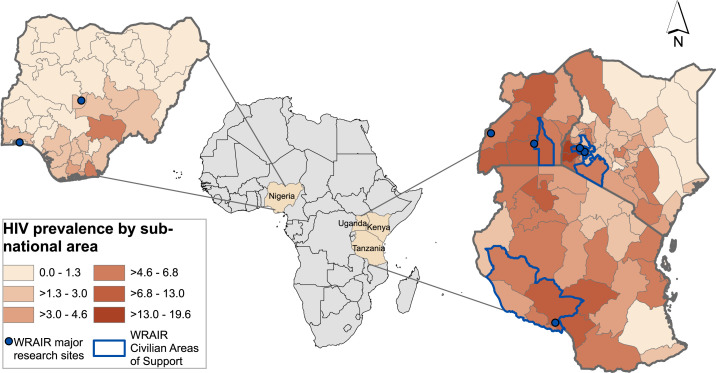
Map of Walter Reed Army Institute of Research’s (WRAIR) President’s Emergency Plan for AIDS Relief (PEPFAR) programs in Africa with HIV subnational prevalence. Program locations for civilian programs are depicted for each country where WRAIR supports U.S. PEPFAR programming. Walter Reed Army Institute of Research also supports military programs in Kenya, Nigeria, and Tanzania not depicted here for security, as well as clinical activities and multiple research sites (see Supplemental Table 1). Major research sites are depicted here. Data accessed on August 23, 2020 as follows: Kenya: 2018 Kenya Public Health Impact Assessment preliminary data https://phia.icap.columbia.edu/kenya-preliminary-report/; Nigeria: 2019 Nigerian HIV/AIDS Indicator and Impact Survey data https://naca.gov.ng/nigeria-prevalence-rate/; Tanzania: 2016–2017 Tanzania HIV/AIDS Indicator Survey data https://phia.icap.columbia.edu/tanzania-summary-sheet/; Uganda: 2016–2017 Ugandan Public Health Impact Assessment data https://phia.icap.columbia.edu/uganda-summary-sheet/; Geographic Information System (GIS) shapefiles: https://www.arcgis.com/home/item.html?id=64aff05d66ff443caf9711fd988e21dd&view=list&sortOrder=true&sortField=defaultFSOrder#overview and https://spatialdata.dhsprogram.com/home/. This figure appears in color at www.ajtmh.org.

Recognizing the threat posed to civilian and military populations, the U.S. Department of Defense (DOD) established the U.S. Military HIV Research Program at the Walter Reed Army Institute of Research (WRAIR) in 1986.^6^ Pioneering WRAIR scientists launched a force-wide HIV screening program and developed countermeasures to protect military and civilian populations through education, antiretroviral therapy (ART), and vaccine development. The Walter Reed Army Institute of Research also established an international network of community-based research sites (Supplemental Table 1), leading to notable successes such as the RV144 “Thai Trial” that established proof of concept for vaccine efficacy and set the stage for ongoing trials of next-generation candidate vaccines.^7,8^ Since 2004, the WRAIR has implemented PEPFAR programs among military and civilian communities in Nigeria, Kenya, Tanzania, and Uganda (Figure 1).

In contrast to its partner organization, the DOD HIV/AIDS Prevention Program (DHAPP), which is focused specifically on working with foreign military partners, WRAIR’s PEPFAR program is unique in that its scope of work explicitly includes both military and civilian partner institutions and populations. The DOD Global Health Engagement model emphasizes partner military health and readiness, engagement of partner militaries in research and public health activities, and community engagement.^9^ Walter Reed Army Institute of Research’s PEPFAR programs advance the PEPFAR goal of epidemic control while adhering to the ethical mandates of equipoise and engagement of multiple stakeholders in clinical research related to its mission of developing vaccines and other countermeasures to HIV. The Department of Defense HIV/AIDS Prevention Program implementation science efforts have centered on seroprevalence and behavioral epidemiology risk surveys and prevention interventions specific to military service members and their families. Although DHAPP-led programs are outside the scope of this article, WRAIR PEPFAR programming benefits from these learning models, and uses implementation science that is responsive to the needs of the diverse population of people and communities served. Although WRAIR’s PEPFAR portfolio is more narrowly focused from a country count perspective than that of the DHAPP, WRAIR programs work on a large scale with high-burden communities, including key and priority populations, using relevant programmatic and implementation science strategies. These continuously evolving programs address the HIV security challenge by improving biopreparedness in alignment with the 2019 United States’ Global Health Security Strategy.^10^

The Walter Reed Army Institute of Research implements research and evaluation efforts at its PEPFAR program sites according to the three components of PEPFAR’s implementation science framework: monitoring and evaluation, operations research, and impact evaluation, to ensure evidence-informed decision-making for program impact and resource allocation.^11^ Implementation science is the study of methods to improve the uptake, implementation, and translation of research findings into routine practices to accelerate progress.^11^ Over the past 15 years, the WRAIR has leveraged its research mission and capabilities to implement an implementation science portfolio to improve HIV service delivery for PEPFAR-supported programs. At this important milestone, we review impacts of this portfolio on HIV programs, policy, and science.

## CONTRIBUTIONS AND IMPACT OF WALTER REED ARMY INSTITUTE OF RESEARCH’S IMPLEMENTATION SCIENCE PORTFOLIO

We reviewed the WRAIR PEPFAR research portfolio from 2003 to 2019, inclusive of PEPFAR-funded studies and externally funded studies that leverage WRAIR’s PEPFAR program sites. We reviewed protocols and publications, and solicited additional information from collaborators and study investigators (Supplemental Tables 2 and 3).

Over the past 15 years, the WRAIR has undertaken 25 PEPFAR-funded implementation science studies, in addition to multiple externally funded studies leveraging PEPFAR sites (Table 1). Of these, 12 have completed clinical data collection, whereas the remainder are in development or ongoing. Walter Reed Army Institute of Research’s implementation science studies have been designed in response to PEPFAR technical priorities in the areas of HIV prevention (*n* = 10); first 95 (*n* = 17), second 95 (*n* = 15), third 95 (*n* = 12); and comorbidities (*n* = 11), with most cross-cutting (*n* = 19). Using this framing, we synthesize studies of chief importance (Table 2). As most people served by WRAIR’s PEPFAR program sites are civilians with no military affiliation, its implementation science research reflects broadly applicable PEPFAR priorities and clinical care.

**Table 1:** Summary of objectives, study designs, and time lines of Walter Reed Army Institute of Research’s PEPFAR-related implementation science protocols

MHRP #/clinical trials #/ACTG#	Title and primary objective	Design*	Duration	WRAIR country
WRAIR Implementation Science Studies with PEPFAR-funding				
RV257/NCT01791556	**Title:** Clinic-based ART Diagnostic Evaluation	Randomized controlled trial	2010–2015	KE, NG, TZ, UG
	**Objective:** To evaluate the superiority and cost-effectiveness of two recommended ministry of health ART diagnostic evaluation approaches at the clinic level in adult treatment-naive patients in routine care, and, viral load–guided care.			
	**Key findings:** A randomized control trial can be enrolled successfully in rural, non-research, resource-limited, district-level clinics in western Kenya. Many adults presenting for ART have advanced HIV/AIDS, emphasizing the importance of universal HIV testing and linkage-to-care campaigns.			
RV288	**Title:** A Virological Assessment of Patients on ART in the US Military HIV Research Program/PEPFAR-Supported Programs in Africa	Cross-sectional	2012–2018	KE, NG, TZ, UG
	**Objective:** To evaluate how to optimize the effectiveness of MHRP-supported antiretroviral treatment programs by identifying program characteristics that result in the best program outcomes and have the greatest impact on reducing treatment failure as defined by viral suppression.			
	**Key findings:** High virological suppression rates are achievable on first-line ART. The odds of virological suppression were positively associated with improvements in CD4 cell percentage and total lymphocyte count, and negatively associated with the cost of travel to the clinic.			
RV292	**Title:** A Point Prevalence Assessment of HIV-1 TB and Malaria Among the Kenyan Military Population	Cross-sectional	2016–2017	KE
	**Objective:** To estimate the prevalence of HIV-1, HIV/TB coinfection rate, and malaria parasitemia among the Kenyan military population.			
	**Key findings:** Kenya Defence Forces received detailed prevalence estimates for HIV-1, HIV/TB coinfection rate, and malaria parasitemia. Risk factor analysis informs both force health protection policy and public health programming.			
RV329	**Title:** African Cohort Study	Cohort	2013–present	KE, NG, TZ, UG
	**Objective:** To longitudinally assess the impact of clinical practices, biological factors and sociobehavioral issues on HIV infection and disease progression in an African context.			
	**Key findings:** Documented decrease in interval between HIV diagnosis and ART initiation from a median of 22 months before 2006 to just 0.5 months after 2016. In addition, found both pretreatment and acquired resistance across the four countries of enrollment, the latter suggesting that strategies emphasizing adherence counseling while delaying ART switch may need modification. Participants with persistent low-level viremia (200–999 c/mL) were more likely to exhibit subsequent virologic failure than those with < 200 c/mL. These findings helped inform downward revision of Tanzanian and Kenyan national viral load threshold definitions of undetectable viral load to < 50 c/mL and < 400 c/mL, respectively.			
RV342	**Title:** A Cost-Outcomes Analysis of the Provision of ART at MCH Centers through 18 Months Post-Partum and then Referral to a General Adult ART Clinic	Cohort	2013–2018	KE
	**Objective:** To evaluate the health facility costs of the provision of ART to ART-eligible pregnant women and evaluate if these women successfully complete the transition from the maternal child health clinic to the HIV clinic for continued ART and care.			
	**Key findings:** The annual incremental cost per patient of providing ART were similar in MCH and non-MCH (ART) clinic settings in 2012. With shifts in recommended ARV regimens and laboratory monitoring over time, annual costs of care (using 2016 USD unit costs) have remained relatively constant in nominal terms for the MCH clinic group but have fallen substantially for the ART clinic group.			
RV346	**Title:** A Cost and Outcomes Analysis of Two Service Delivery Models of Safe Male Circumcision in Rural Uganda	Micro-costing	2012	UG
	**Objective:** To estimate the average cost of performing VMMCs in the mobile clinic compared with those performed in health facilities (fixed sites).			
	**Key findings:** The Makerere University Walter Reed Project VMMC program improves access for hard to reach, relatively poor, and high-risk rural populations for a cost of $27–$38 per circumcision. Costs to access services are almost certainly less in the mobile program, by reducing out-of-pocket barriers such as travel expenses and lost time and associated income.			
RV399	**Title:** Evaluating the HIV Infant Tracking System (HITSystem) to Improve Infant Diagnosis of HIV in the US Military HIV Research Program/PEPFAR in the Southern Highlands Using the HITSystem	Cohort	2017–2018	TZ
	**Objective:** To evaluate the HIV Infant Tracking System’s capacity to improve the 1) retention and 2) timely provision of time-sensitive intervention benchmarks for early infant diagnosis services and outcomes.			
	**Key findings:** Improvements were seen in linkage to care, retesting of infants, tracking of sample collection and transport with associated turnaround time, and initiation of infants living with HIV on treatment, leading to increased early infant diagnosis service uptake and improved infant outcomes.			
RV415	**Title:** Evaluation of Changing Guidelines, Systems and Practices on Prevention, Care and Treatment Activities in the Buvuma, Kayunga, and Mukono Districts in Uganda	Various, evaluation	2015–present	UG
	**Objective:** To assess effects of clinical, operational, sociobehavioral, and regulatory factors on prevention, care, and treatment programs in Uganda.			
	**Key findings:** Multiple analyses in progress.			
RV433	**Title:** Monitoring HIV Drug Resistance Using WHO Early Warning Indicators in the Southern Highlands of Tanzania	EWI1, EWI4: cross-sectional; EWI2, EWI3, EWI5: cohort	2015–2018	TZ
	**Objective:** To determine HIV drug resistance early warning indicators in the Southern Highlands’ care and treatment centers to evaluate the quality of ART services uptake with an aim of helping to minimize the emergence of preventable HIV drug resistance. EWIs include EWI1: on-time pill pick-up by patients at the site pharmacy; EWI2: patient retention on treatment after 1 year of ART; EWI3: pharmacy stock-outs of ARV drugs each month during a period of 1-year; EWI4: ARV dispensing practices in mono-, bi-, or triple-ART known as highly active ART; EWI5: viral load suppression at 12 months.			
	**Key findings:** More than 90% of clinics in Southern highlands performed relatively well in terms of dispensing practices (EWI4). However, ART facilities underperformed (< 75%) in all other indicators, suggesting a high chance of emergence of HIV drug resistance among PLHIV. There is a need for continuous monitoring of drug resistance to ensure high performance (EWI1-3).			
RV432	**Title:** Improving Retention in HIV Care and Treatment Services through the Development of a Network of ART Clinics within the Fishing Communities on Koome Island, Uganda.	Phase 1: cross- sectional; phase 2: cohort	2016–2019	UG
	**Objective:** To evaluate the impact of the meta-ART clinic network approach on the rate of retention in HIV clinical services among fisherfolk on Koome Island.			
	**Key findings:** HIV is highly prevalent in the Koome and Buvuma fishing communities, well above the 6.2% national prevalence. The fishing community is a heterogeneous population with varied characteristics and different HIV and STI risk profiles. Higher than national average of HIV and syphilis prevalence calls for intensified HIV/STI prevention efforts.			
RV449	**Title:** Evaluation of Implementing Guidelines, Systems, and Best Practices on HIV Prevention, Care, and Treatment Activities in Mbeya, Rukwa, Ruvuma, Katavi, Njombe and Iringa Regions, United Republic of Tanzania	Various, evaluation	2017–present	TZ
	**Objective:** To assess the effects of clinical, operational, sociobehavioral, and regulatory factors on prevention, care, and treatment programs.			
	**Key findings:** Multiple analyses in progress. Linkage case management is a successful strategy for active linkage and early retention among adult PLHIV and is likely to improve program performance if implemented in other PEPFAR countries. Programs should expand LCM to children living with HIV and their caregivers, as well as previously diagnosed individuals not yet linked to care and treatment; expand the duration of case management to 6 months or until viral suppression; increase the number of linkage case managers; and offer regular refresher training.			
RV465	**Title:** Implementing the PMTCT Standard of Care under Routine Conditions with and without the EMMA: A Site-Randomized Impact Evaluation Study among Maternal and Child Health Clinics Supported by the South Rift Valley PEPFAR Program in Kenya	Cluster randomized controlled trial	2015–2019	KE
	**Objective:** To evaluate impacts on key adherence and retention outcomes of the EMMA strategy to improve implementation of the standard of care and estimate the additional cost to the PMTCT program of implementing the EMMA strategy.			
	**Key findings (unpublished]:** Continuous ART coverage during two periods of the PMTCT care cascade was low between EMMA and standard of care groups.			
RV476	**Title:** HIV PrEP among HIV Negative Adolescent Girls and Young Women Sex Workers, Aged 18–24 Years Mukono District, Uganda: A Pragmatic Demonstration Project.	Cohort	2017–2019	UG
	**Objective:** To assess the feasibility of implementing PrEP in Uganda among adolescent girls and young women sex workers by evaluating 1) acceptability, 2) uptake of PrEP, 3) adherence to PrEP, and 4) retention rates in PrEP programs.			
	**Key findings:** Regardless of time on PrEP, three reasons for PrEP discontinuation included side effects, life transitions, and disclosure. Programs need to provide more supportive counseling to help manage the side effects associated with PrEP and increase uptake. Counseling sessions need to address how to maintain HIV risk reduction strategies during key life transitions. Furthermore, PrEP packaging needs to be easily differentiated from ART packaging, and PrEP messaging should aim to create broader awareness to reduce stigma.			
RV517	**Title:** Prevalence of, and Factors Associated with, Virologic Suppression and Drug Resistance in HIV Positive Children and Adolescents on ART in Tanzania	Cross-sectional	2019–present	TZ
	**Objective:** To optimize the effectiveness of MHRP-supported antiretroviral treatment programs by identifying program characteristics that result in the best program outcomes and have the greatest impact on reducing treatment failure in children and adolescents as defined by viral suppression.			
	**Key findings:** Data collection in progress.			
RV518	**Title:** Prevalence of, and Factors Associated with, Virologic Suppression and Drug Resistance in HIV Positive Children and Adolescents on ART in Kenya	Cross-sectional	2019–present	KE
	**Objective:** To optimize the effectiveness of MHRP-supported antiretroviral treatment programs by identifying program characteristics that result in the best program outcomes and have the greatest impact on reducing treatment failure in children and adolescents as defined by viral suppression.			
	**Key findings:** Data analysis in progress.			
RV528	**Title:** WHO “Test and Start” Strategy and Programmatic Implications for TB-HIV in Nigeria: An Evaluation of a Treatment Program in 27 Nigerian Military Hospitals	Pre- and post-evaluation	2018–present	NG
	**Objective:** To evaluate the impact of the current WHO “Test and Start” strategy on the TB care cascade in PEPFAR supported TB-HIV programs.			
	**Key findings:** TB preventive therapy initiation increased during the “surge” and plateaued during “sustained” phases. Because of an isoniazid stock-out, a “dip phase” was recorded. There was overall initiation rate of 79%. Completion rates were, respectively, 73% and 70% for FY2018 and FY2019.^54^			
RV543	**Title:** Evaluation of Changing Guidelines, Systems, and Practices on Prevention, Diagnostic, and Treatment Activities in the Military HIV Program in Nigeria	Various, evaluation	2019–present	NG
	**Objective:** To assess the effects of clinical, operational, sociobehavioral, and regulatory factors on prevention, care, and treatment programs.			
	**Key findings:** Multiple analyses in progress.			
RV553	**Title:** HIV-1 Recent Infection Surveillance among Persons Newly Diagnosed with HIV Infection in Kenya	Various, surveillance	In review	KE
	**Objective:** To establish systems for continuous epidemiological surveillance for recent HIV infection using data on person, place, and time of newly diagnosed individuals to inform HIV prevention and treatment strategies, and to contribute to understanding of HIV transmission dynamics.			
	**Key findings**: Data collection not yet initiated for WRAIR sites.			
RV555	**Title:** Taking TB Preventive Therapy to a National Scale: The Nigeria PEPFAR Program Experience	Pre- and post-evaluation	2019–2020	NG
	**Objective:** To assess the level of TB preventative treatment scale-up across PEPFAR-Nigeria HIV-supported programs, and to evaluate the effects of program and policy changes on the implementation of preventative treatment for the FY 2015–2017 period.			
	**Key findings:** An increasing trend in TPT uptake between the pre- and post-intervention periods was observed: 6% in FY 2015, 7% in FY2016, and 12% in FY2017. Logistical changes to include INH in 2016 led to a statistically significant 69% increase in TPT by the end of FY2017.^55^			
RV556	**Title:** HIV-1 Recent Infection Surveillance among Persons Newly Diagnosed with HIV Infection in Uganda	Various, surveillance	In review	UG
	**Objective:** To establish systems for continuous epidemiological surveillance for recent HIV infection using data on person, place, and time of newly diagnosed individuals to inform HIV prevention and treatment strategies, and to contribute to understanding HIV transmission dynamics.			
	**Key findings:** Data collection not yet initiated at WRAIR sites.			
RV557	**Title:** TB Prevention, Diagnostic and Treatment Cascade Process Evaluation	Post-intervention evaluation	2019–2020	NG
	**Objective:** To evaluate the effects of program policy changes and of supportive interventions on the implementation of TB preventative treatment and optimization of GeneXpert MTB/Rif assay testing for TB, as well as on TB patient treatment retention, treatment completion, and associated outcomes.			
	**Key findings:** Analysis in progress.			
RV558	**Title:** HIV-1 Recent Infection Surveillance among Persons Newly Diagnosed with HIV Infection in Tanzania	Various, surveillance	In review	TZ
	**Objective:** To establish systems for continuous epidemiological surveillance for recent HIV infection using data on person, place, and time of newly diagnosed individuals to inform HIV prevention and treatment strategies, and to contribute to understanding HIV transmission dynamics.			
	**Key findings:** Data collection not yet initiated at WRAIR sites.			
RV559	**Title:** Improving Viral Load Suppression in Adolescents and Young Adults 10–24 Years Using Adolescent Friendly Services	Post-intervention evaluation	2019–present	NG
	**Objective:** To assess the effect of a package of focused adolescent-and youth-friendly services initiated at NMOD health facilities in partnership with the U.S. MHRP and the U.S. Army Medical Research Directorate-Africa/Nigeria on key aggregate clinical outcomes tracked at the facility level.			
	**Key findings (unpublished]:** The implementation of intensive, patient-centered, individual, and group interventions for HIV-positive adolescents is critical to keeping them virally suppressed and reducing onward transmission. Healthcare providers will need to commit time and effort to making adolescents services visible, flexible, affordable, confidential, and culturally appropriate. Intensive, site-, and patient-level monitoring is required to ensure VL suppression.			
RV561	**Title:** HIV-1 Recent Infection Surveillance among Persons Newly Diagnosed with HIV Infection in Nigeria	Various, surveillance	In review	NG
	**Objective:** To establish systems for continuous epidemiological surveillance for recent HIV infection using data on person, place, and time of newly diagnosed individuals to inform HIV prevention and treatment strategies, and to contribute to understanding of HIV transmission dynamics.			
	**Key findings:** Data collection not yet initiated at WRAIR sites.			
RV563	**Title:** Routine Program Evaluation of HIV Prevention, Diagnostic, and Treatment Services in Kenya to Inform Evidence-Based Improvements in Programs and Policies	Various, evaluation	2019–present	KE
	**Objective:** To evaluate PEPFAR supported programs in the areas of HIV, TB, and other associated disease in settings of relevance to the Kenya Ministry of Health, with the overall goal of contributing to evidence-informed decision-making and improvements to ongoing and future interventions designed to move Kenya toward epidemic control.			
	**Key findings:** Multiple analyzes in progress.			
Select WRAIR Implementation Science Studies with outside funding that leverage PEPFAR sites				
RV186/NCT00089505/A5208	**Title:** Optimal Combination Therapy After Nevirapine Exposure	Open-label, randomized intervention	2011–2018	KE
	**Objective:** To compare the efficacy of NNRTI- and PI-containing anti-HIV regimens in women who have previously taken NVP for MTCT of HIV and in women who have never taken NVP. To monitor, in greater extent, the participants’ health as they transition from study treatment to local, clinical care.			
	**Key findings**: In women with prior exposure to peripartum single-dose nevirapine (but not in those without prior exposure), ritonavir-boosted lopinavir plus tenofovir–emtricitabine was superior to nevirapine plus tenofovir–emtricitabine for initial ART.^50^			
RV246/NCT00286767	**Title:** A Cohort Observational Study Evaluating Predictors, Incidence and IRIS in HIV-1 Infected Patients with CD4 Count Less Than or Equal to 100 Cells/µL Who Are Initiating ART	Cohort	2006–2008	KE
	**Objective:** To evaluate the predictors, incidence, clinical presentation, and IRIS in HIV-1–infected patients with CD4 count less than or equal to 100 cells/microL who are initiating ART.			
	**Key findings:** For PLHIV with severe immunosuppression initiating ART, baseline low body mass index and hemoglobin, and high CRP and D-dimer levels may be clinically useful predictors of IRIS and death risk.^24^			
RV249/NCT00108862/A5221	**Title:** Immediate Versus Deferred Start of Anti-HIV Therapy in HIV-Infected Adults Being Treated for TB (STRIDE)	Open-label, randomized intervention	2006–2010	KE
	**Objective:** To determine the best time to begin anti-HIV treatment in individuals who have HIV and TB.			
	Key findings: Early ART in HIV-infected adults with newly diagnosed TB improves survival in those with CD4^+^ T-cell counts less than 0.050 × 109 cells/L, although this is associated with a 2-fold higher frequency of TB-immune reconstitution inflammatory syndrome. In patients with CD4^+^ T-cell counts greater than 0.050 × 109 cells/L, evidence is insufficient to support or refute a survival benefit conferred by early vs. delayed ART initiation.^56^			
**RV367/NCT01404312/A5279**	**Title:** Brief Rifapentine-Isoniazid Evaluation for TB Prevention	Open-label, randomized intervention	2012–2017	KE
	**Objective:** To compare the safety and effectiveness of a 4-week daily regimen of RPT plus INH to a standard 9-month daily INH regimen for TB prevention in HIV-infected individuals.			
	**Key findings**: A 1-month regimen of rifapentine plus isoniazid was noninferior to 9 months of isoniazid alone for preventing TB in HIV-infected patients. The percentage of patients who completed treatment was significantly higher in the 1-month group.^49^			
RV368	**Title:** HIV and STI Prevalence, Incidence and Risk Behaviors among Men Who Have Sex with Men (MSM) in Nigeria and Impact of Providing HIV Medical and Prevention Services to Nigerian MSM at a Trusted Community Center (TRUST)	Cohort	2013–2018	NG
	**Objective:** To evaluate the impact of provision of comprehensive and integrated prevention, treatment, and care at a community-based clinic operated by an organization experienced in providing HIV prevention, care, and support services to key populations.			
	**Key findings:** Multiple analyses complete or in progress. Although there was high acceptance of HIV testing and low loss to follow-up among individuals who were already on ART or engaged in treatment as prevention (TasP), a higher than expected proportion did not engage in TasP, suggesting the need for customized treatment preparation and an increase in enabling environments to support HIV treatment access with this key population.^57^			
RV494/NCT02891135	**Title:** Randomized Evaluation of a Simplified Clinical Algorithm for Identifying Patients Eligible for Immediate Initiation of ART for HIV (SLATE)	Open-label, randomized intervention	2016–2017	KE
	**Objective:** To determine the effectiveness of the SLATE algorithm in increasing ART initiation, compared with standard care, in nonpregnant adults.			
	**Key findings:** In South Africa, the SLATE algorithm increased the uptake of ART within 28 days by 10% and showed a numerical increase (6%) in retention at 8 months. In Kenya, the algorithm increased the uptake of ART within 28 days by 6% but found no difference in retention at 8 months. Eight-month retention was poor in both arms and both countries. These results suggest that a simple structured algorithm for same-day treatment initiation procedures is feasible and can increase and accelerate ART uptake but that early retention on treatment remains problematic.^22^			
RV516	**Title:** Impact Evaluation of PEPFAR-funded PMTCT Activities on Infant Mortality in Kenya	Pooled cross-sectional	2016–2018	KE
	**Objective:** To evaluate the impact of PEPFAR funding on key PMTCT related health outcomes, including infant mortality, in Kenya and to assess the degree to which the relationship between PEPFAR funding and key PMTCT-related health outcomes is mediated by process indicators collected as part of the routine monitoring and evaluation of PEPFAR-funded PMTCT programs.			
	**Key findings:** Evidence from publicly available data suggests that PEPFAR’s PMTCT funding was associated with a reduction in infant mortality and an increase in HIV testing during ANC in Kenya. The full outcome of funding may not be realized until several years after allocation.^17^			

ACTG = AIDS Clinical Trials Group; ANC = antenatal care; ART = antiretroviral therapy; EMMA = Enhanced Mentor Mother progrAm; EWI = early warning indicator; FY = fiscal year; INH = isoniazid; IRIS = Immunopathogenesis of Immune Reconstitution Syndrome; KE = Kenya; MCH = maternal and child health; MHRP = Military HIV Research Program; NG = Nigeria; NVP = nevirapine; PEPFAR = President’s Emergency Plan for AIDS Relief; PLHIV = people living with HIV; PMTCT = prevention of mother-to-child transmission; PrEP = Pre-exposure prophylaxis; RPT = rifapentine; TPT = tuberculosis preventive treatment; TZ = Tanzania; TB = tuberculosis; UG = Uganda; VL = viral load; VMMC = voluntary medical male circumcision; WRAIR = Walter Reed Army Institute of Research.

* Design is detailed according to primary objective; additional designs may be used to support analysis of secondary or exploratory objectives.

**Table 2:** Categorization of Walter Reed Army Institute of Research’s PEPFAR-related implementation science protocols by the PEPFAR implementation science framework and priority program areas

#	ID	PEPFAR implementation science framework component*	PEPFAR priority program areas				
			Prevention	First 95 HTS*	Second 95 C&T*	Third 95 VLS*	Comorbidities
WRAIR Implementation Science Studies with PEPFAR-funding							
1	RV257	Impact evaluation	–	–	–	X	–
2	RV288	Surveillance	–	–	–	X	–
3	RV292	Surveillance	–	X	X	–	X
4	RV329	Operations research	X	X	X	X	X
5	RV342	Operations research	X	–	X	–	–
6	RV346	Operations research	X	X	–	–	–
7	RV399	Monitoring and evaluation	X	X	X	–	–
8	RV415	Monitoring and evaluation	X	X	X	X	X
9	RV433	Operations research	–	–	X	X	–
10	RV432	Impact evaluation	–	X	X	X	X
11	RV449	Monitoring and evaluation	X	X	X	X	X
12	RV465	Impact evaluation	X	X	X	–	–
13	RV476	Operations research	X	X	–	–	X
14	RV517	Operations research	–	–	X	X	–
15	RV518	Operations research	–	–	X	X	–
16	RV528	Monitoring and evaluation	–	–	X	–	X
17	RV543	Monitoring and evaluation	X	X	X	X	X
18	RV553	Surveillance	–	X	–	–	–
19	RV555	Monitoring and evaluation	–	X	X	–	X
20	RV556	Surveillance	–	X	–	–	–
21	RV558	Surveillance	–	X	–	–	–
22	RV557	Monitoring and evaluation	–	X	X	–	X
23	RV559	Monitoring and evaluation	–	–	X	X	–
24	RV561	Surveillance	–	X	–	–	–
25	RV563	Monitoring and evaluation	X	X	X	X	X
Select WRAIR Implementation Science Studies with outside funding that leverage PEPFAR sites							
26	RV368	Operations research	X	X	X	X	X
27	RV494	Operations research	–	–	X	–	–
28	RV516	Impact evaluation	X	X	X	–	–
29	RV246/A5221	Operations research	–	X	X	–	X
30	RV367/A5279	Operations research	–	–	X	–	X

C&T = care and treatment; HTS = HIV testing services; ID = study identification number; PEPFAR = President’s Emergency Plan for AIDS Relief; WRAIR = Walter Reed Army Institute of Research; VLS = Viral load suppression.

* Exemplary ACTG trials with major findings/publications.

### Prevention.

Walter Reed Army Institute of Research’s prevention studies address PEPFAR’s focus on key prevention activities for populations at high risk for HIV acquisition: pre-exposure prophylaxis (PrEP), voluntary male medical circumcision, empowerment of adolescent girls and young women, and prevention of mother-to-child transmission (PMTCT) of HIV infection. For example, in Uganda, WRAIR and Makerere University Walter Reed Project (MUWRP) routinely work together to improve implementation of prevention services for priority populations through translation of research findings to programmatic improvements. This partnership assessed acceptability, uptake, and adherence to PrEP, as well as retention in care, among adolescent girls and young women who sell sex, a major behavioral driver of incident HIV infection (RV476).^12,13^ Challenges with stigma and gender-based violence due to misunderstanding of PrEP as ART because of similar packaging were noted and addressed through counseling and referral. Poor PrEP adherence through comparison of serum tenofovir levels to pill counts has also informed counseling during PrEP services (unpublished data). Through the determined, resilient, empowered, AIDS-free, mentored, and safe program, WRAIR and MUWRP have linked these girls to a core packet of interventions to achieve a substantial reduction in new infections among adolescent girls and young women. The evidence from RV476 has been disseminated to key in-country stakeholders and communicated across various international platforms (Supplemental Table 3) to refine PrEP implementation guidelines and further inform PrEP delivery strategies in this key population.

In partnership with the WRAIR, in 2009, MUWRP also instituted the first facility-based voluntary medical male circumcision program in Uganda, followed up by the introduction of a rural community-based mobile clinic in 2011. In collaboration with Boston University, a micro-costing study estimated the cost of improving access to male circumcision in the mobile clinic setting compared with health facilities (RV346). The study found that the mobile clinic program improved access for remote, high-risk, relatively poor populations at a cost of $27–$38 per circumcision through reductions in out of pocket expenditures for travel and lost time at work.^14^ Over time, WRAIR’s programming in East Africa has led to high uptake of male circumcision in civilian and military programs, which creates a useful backdrop for future studies to occur, potentially reducing incident infections. Walter Reed Army Institute of Research’s counterpart DHAPP has also worked extensively with military populations on voluntary medical male circumcision programming and implementation science from the earliest days of this intervention.^15^

Walter Reed Army Institute of Research’s PEPFAR programs implement PMTCT interventions alongside studies of strategies to identify women living with HIV, and protect uninfected mothers and improve delivery of care.^16^ In collaboration with Harvard University, WRAIR and its Kericho site in Kenya helped estimate that 273,924 infant deaths were averted in Kenya between 2004 and 2014 through PEPFAR PMTCT funding, or that a $0.33 increase in per capita PEPFAR PMTCT funding was associated with a 14–16% reduction in infant mortality (RV516).^17^ In partnership with Boston University, the WRAIR explored the impact of adding mentor mothers versus standard care alone on maternal and infant outcomes (RV465), and demonstrated that maternal and child health clinics offered a lower cost alternative for initiating pregnant women on ART (RV342).^18,19^ The Walter Reed Army Institute of Research also evaluated a pilot of the automated HIV Infant Tracking System (HITSystem^©^, Global Health Innovations, Dallas, TX) in the Southern Highlands of Tanzania in 2013 to improve maternal and infant outcomes (RV399). Linkage to care, retesting of infants at critical time points, tracking of sample collection and transport with associated turnaround time, and initiation of infants living with HIV on treatment improved, leading to increased early infant diagnosis service uptake and improved infant outcomes overall. The Tanzanian Ministry of Health and the National AIDS Control Program recognize these features as a best practice and are considering broader implementation.

### The first 95: testing.

As of 2018, approximately 21% of PLHIV worldwide were unaware of their status.^2^ President’s Emergency Plan for AIDS Relief makes HIV testing services freely available to address this gap and achieve the UNAIDS goal for 95% of PLHIV to know their status by 2030.^20^ The Walter Reed Army Institute of Research routinely evaluates program testing performance across testing modalities at facilities and in communities disaggregated by age-group, gender, and key populations, with special emphasis on adolescents, young adults, and adult men.

HIV surveillance studies help to identify and close the remaining gaps to reach the first 95 in specific populations. In 2015, the WRAIR collaborated with the Kenya Defense Forces to conduct a prevalence assessment of HIV, tuberculosis (TB), and malaria among the Kenyan military population (RV292). This study helped the WRAIR and the Kenyan military to focus testing initiatives on highest risk populations within the forces. Furthermore, findings informed testing modality prioritization and planning for force healthcare needs and contributed to improved case finding in men and the services generally. In Uganda, the WRAIR conducted an integrated behavioral and biological survey, finding a high prevalence of HIV, hepatitis B, and seropositivity for syphilis in remote fishing communities on Koome and Buvuma islands, while linking newly identified participants to treatment (RV432) (unpublished data).

Recently, many countries have undertaken surveillance activities to estimate HIV prevalence, assess progress toward 95-95-95, and inform resource allocation for targeted case finding.^21^ Surveillance systems incorporating rapid tests for recent infection within the past year are supported by the WRAIR and other PEPFAR implementing agencies in Kenya, Nigeria, Tanzania, and Uganda. Data from these studies will inform the incorporation of recency testing into national surveillance activities for early detection of transmitting networks, such as during outbreak investigations. Each of these examples of WRAIR’s contribution to surveillance provides critical information for targeting programmatic response in the highest risk populations.

### The second 95: treatment.

Through multiple studies, WRAIR has addressed improvements in care for the second 95, which aims for PLHIV to initiate and adhere to lifelong, uninterrupted ART. In collaboration with Boston University, WRAIR-supported PEPFAR sites in Kericho, Kenya, evaluated an algorithm to determine eligibility for same-day initiation of ART, with an overall goal of reducing patient attrition at point of care (RV494).^22^ The trial found persistent barriers to early or same-day ART initiation based on clinical practice,^23^ highlighting the need for improved guidance and training for clinical providers. The site also collaborated with the NIH, finding that for PLHIV with severe immunosuppression who initiate ART, baseline low body mass index (BMI) and hemoglobin with high C-reactive protein and D-dimer may be clinically useful predictors of immune reconstitution inflammatory syndrome and death (RV246).^24^

One of the largest and longest running protocols under WRAIR’s PEPFAR research portfolio with second 95 impacts is The African Cohort Study (AFRICOS). The African Cohort Study examines the drivers of quality HIV clinical care across WRAIR programs in four countries, fosters international and domestic partnerships, enables ongoing evaluation of PEPFAR performance, and elucidates ART as a chronic care model within resource-constrained settings (RV329). Consistent with changing guidelines for ART initiation, the study has documented a decreased interval between HIV diagnosis and ART initiation from a median of 22 months before 2006 to just 0.5 months after 2016.^25^ As millions of PLHIV transition to dolutegravir-based ART, AFRICOS is poised to track health-related outcomes including development of comorbid conditions, adverse effects, and drug resistance. In response to growing concern about HIV prevention and treatment in young age-groups, the study has recently been expanded to include participants aged 15–17 years, to inform programs and policies for this critical age-group with historically poor HIV-related outcomes.

The African Cohort Study has also leveraged data to describe gender-based differences in HIV care and treatment to bridge critical gender-specific gaps. Despite experiencing more social barriers to care, women are diagnosed and engaged in care earlier in their disease process compared with men, partly because of availability of joint women-centric services at HIV clinical facilities such as prenatal and reproductive health services (unpublished). Differentiated models of care delivery are needed to improve engagement of men and optimize support to already-engaged women. In addition, data from AFRICOS have shown that advanced HIV disease at enrollment, defined as CD4 < 200 cells/mm^3^, was more common among participants who were in Tanzania, male, aged > 29 years, highly educated, and with higher WHO clinical stage. Factors associated with a lower risk of advanced disease were > 1 year since HIV diagnosis and being on ART for at least 6 months (unpublished). These results have informed targeting of individual factors in WRAIR-supported PEPFAR programs to address advanced disease when refining care and treatment strategies, especially in the era of test and treat.

### The third 95: viral suppression.

Attaining viral suppression requires a combination of the right drug regimen, medication adherence, and viral load monitoring, and WRAIR has been at the forefront of this work. In 2010, WRAIR undertook the Clinic-based ART Diagnostic Evaluation randomized, controlled trial in Kenya that demonstrated the feasibility, superiority, and cost-effectiveness of routine viral load versus standard of care monitoring in adults initiating ART at that time in rural, district-level HIV clinics (RV257).^26^ The clinic-based ART Diagnostic Evaluation reflected WRAIR’s forward posture on optimal patient monitoring, and findings supported global policy updates made shortly thereafter for routine viral load monitoring.^27^ The Clinic-based ART Diagnostic Evaluation demonstrated that rigorous trials could be conducted in a routine clinical setting, paving the way for future, generalizable research including the AFRICOS.

Walter Reed Army Institute of Research has also evaluated early signs of population-level drug resistance and assessed viral load and drug resistance patterns in children and adults (Table 1). In 2016, WRAIR undertook a retrospective evaluation of early warning indicators of drug resistance in the Southern Highlands of Tanzania (RV433), which improved the capacity of healthcare providers to conduct surveillance for drug resistance and informed the 2019 National Report for Monitoring Development of HIV Drug Resistance.^28^ More recently, AFRICOS found both pretreatment and acquired resistance across the four countries of enrollment, the latter suggesting that strategies emphasizing adherence counseling while delaying ART switch may need modification.^29^ In collaboration with the Uniformed Services University of the Health Sciences, viral load and drug resistance data from children aged 1–19 years in Tanzania (RV517) and Kenya (RV518) are being used to inform real-time treatment decisions for regimen optimization. Forthcoming results will inform funding and policy decisions.

African Cohort Study findings support a growing body of literature that suggests the accepted viral suppression threshold may need to shift from 1,000 to a more stringent < 200 copies per milliliter (c/mL). African Cohort Study participants with persistent low-level viremia (200–999 c/mL) were more likely to exhibit subsequent virologic failure than those < 200 c/mL.^30^ These findings helped inform downward revision of Tanzanian and Kenyan national viral load threshold definitions of undetectable viral load to < 50 c/mL and < 400 c/mL, respectively.^31,32^ The Nigeria program is using this information to enroll clients with persistent, low-level viremia into enhanced adherence counseling with repeat viral load testing to achieve suppression < 200 c/mL. In the era of Undetectable = Untransmittable, viral load threshold policy refinements have the potential to prevent significant transmission of the virus.

### Comorbidities.

President’s Emergency Plan for AIDS Relief strategies for implementing a multipronged approach to HIV epidemic control include prevention and clinical services for HIV-related comorbidities such as TB, sexually transmitted infections, and mental health disorders. In response, WRAIR has incorporated each of these comorbidities into implementation science efforts. For example, the program in Kericho, Kenya, supported the first regional hospital to integrate TB and HIV care in a single clinic in 2005.^33^ In addition, WRAIR has conducted several evaluations of TB and TB preventive therapy in Nigeria, which have been critical in direction setting for the Nigerian Ministry of Defence program (RV528 and RV557) and nationally (RV555). Preliminary data have led to expanded GeneXpert laboratory services (Sunnyvale, CA) and collaborations, improved sample referral and operations, and increased uptake of TB preventive therapy among PLHIV in Nigeria.

The African Cohort Study characterizes how HIV comorbidities such as malignancy, cardiovascular events, malnutrition, anemia, and cognitive decline impact clinical outcomes and help define the healthcare needs of an aging African HIV population. Cervical cancer accounts for 25% of female cancers in Africa, with an incidence of 31 per 100,000 women.^34,35^ African Cohort Study data on cervical cancer screening help set PEPFAR program targets and will further elucidate the interplay between HIV, the immune system, and human papillomavirus–induced carcinogenesis.^36^ African Cohort Study findings suggest that PLHIV on ART had an increased risk of noncommunicable diseases compared with those not on ART.^37^ In addition, AFRICOS is being used to monitor the transition to dolutegravir as first-line treatment, providing real-time data to inform program improvement. Preliminary data show that participants prescribed tenofovir/lamivudine/dolutegravir (TLD) developed a higher BMI than those on other ART regimens (unpublished). Participants who were ART naive had a 55% lower rate of developing high BMI than those on non-TLD ART, and hyperglycemia incidence was increased with exposure to any ART, particularly TLD (unpublished). These findings have policy implications for rollout of dolutegravir, as metabolic adverse event profiles differ between regimens. Comprehensive care will need to address noncommunicable diseases in PLHIV as they survive to older ages.^37^ The cohort has also identified mental health as a driver of HIV outcomes, for example, describing depression as a predictor of HIV viral load.^38^

### Leveraging PEPFAR sites for impact.

Walter Reed Army Institute of Research leverages knowledge and skills in PEPFAR program management, research, and implementation science for translation of research into impact. Walter Reed Army Institute of Research’s PEPFAR research portfolio incorporates institutional partnerships and synergies that maximize return on investments. Collaborations with other U.S. government agencies and academic partners such as Boston University, Harvard University, and University of Maryland have leveraged WRAIR’s PEPFAR sites and external funding to conduct implementation science work to answer key service delivery questions.

The TRUST study, supported by DOD and NIH funding, used respondent-driven sampling to enroll highly marginalized men who have sex with men and transgender women at PEPFAR sites into HIV programs at community centers in Nigeria (RV368). The study identified an exceptional burden of HIV in Nigerian men who have sex with men, with HIV prevalence more than 50% and incidence more than 15 cases per 100 person-years.^39,40^ The study also identified a substantial need for diagnosis and management of other sexually transmitted infections, and new prevention interventions.^40–47^ Study findings have subsequently been incorporated directly into prevention and clinical service approaches at the community centers.

In Kericho, Kenya, a WRAIR- and PEPFAR-supported clinical care center is co-located with an AIDS Clinical Trials Group clinical research site. Two major publications in *The New England Journal of Medicine* using data from this site resulted in global policy changes, including shifting standard of care for starting ART while on TB treatment from 6 months to 2 weeks of TB therapy in patients with CD4 < 50 cells/mm^3^ (RV249).^48^ The site also contributed to the groundbreaking Brief Rifapentine–Isoniazid Evaluation for TB Prevention/A5279 trial that established the efficacy of a 1-month rifapentine/isoniazid regimen for the prevention of active TB and provided data to guide future TB/HIV program activities (RV367).^49^ In 2006, the site collaborated with the NIH in the OCTANE/A5208 study (RV186). This study demonstrated ritonavir-boosted lopinavir plus tenofovir-emtricitabine was superior to nevirapine plus tenofovir-emtricitabine for initial ART in women with prior use of single-dose nevirapine, and that clinical trial adult female participants could successfully be transitioned to local, routine care.^50,51^ The site has further collaborated with the NIH to conduct studies in infants, children, adolescents, and pregnant women to improve HIV treatment and prevention including HIV remission. By implementing collaborations with access to diverse funding streams, WRAIR has created additional opportunities to build upon a strong foundation to translate research into public health impact.

### Biopreparedness.

President’s Emergency Plan for AIDS Relief investments in Africa have helped build a strong foundation for biopreparedness and health security through systems that strengthen local capacity and emphasize effective, efficient, and sustainable health care.^52^ These long-term public health and health system strengthening tools have been applied to emerging infectious disease threats, including the 2014 Ebola outbreaks in Nigeria and Uganda and the current COVID-19 pandemic.^53^ Walter Reed Army Institute of Research is strongly positioned to rapidly respond to such outbreaks through clinical research and implementation science, in addition to ongoing diagnostic and vaccine development lines of effort. Research skills developed in the conduct of trials and observational studies lay the foundation for public health and countermeasure development across sub-Saharan Africa.

### Data for public health impact.

Walter Reed Army Institute of Research’s program evaluations integrate core performance data streams continuously analyzed across all PEPFAR programs, with operational and research data collected at the service delivery level to identify program strengths and gaps. For example, Nigerian program direction has been informed by improvements in adolescent viral suppression observed at Nigerian military sites after implementation of a strategy originally piloted in Kenya (RV543). Program evaluations improve staff capacity to critically assess program outputs against the 95-95-95 targets and implement evidence-informed strategies in response to real-time programming needs. Similarly, AFRICOS data are leveraged to assess PEPFAR priorities, providing real-time feedback to inform program improvement. This includes current priorities such as monitoring the transition to dolutegravir as first-line treatment, the transition to multi-month ART dispensing, viral load suppression and HIV drug resistance, and HIV comorbidities and advanced disease. As PEPFAR countries approach epidemic control, finding cases and maintaining clients on care long-term require increasingly granular data and rapid analysis. Chronic care models will become increasingly important in the care and treatment setting, as will data on HIV coinfections and comorbidities. Longitudinal studies embedded in PEPFAR program sites, such as AFRICOS, present a unique opportunity to characterize these conditions among adolescent and adult populations.

## CONCLUSION

Walter Reed Army Institute of Research’s diverse implementation science portfolio has had a significant impact on HIV prevention, 95-95-95, and comorbidity-focused programs, and has informed policies for care delivery across PEPFAR priority areas. Impacts include improved PrEP delivery and PMTCT strategies; results return, linkage, and clinical management; expansion of viral load testing, especially in key and priority populations; and medication adherence through viral load monitoring. In addition, the portfolio addresses the complete spectrum of long-term HIV health, including comorbidities as the population ages.

Nested within the WRAIR enterprise, investments in implementation science provide direct benefit to PEPFAR service delivery for military and civilian populations while creating institutional synergies that contribute broadly to the DOD’s global health-oriented research mission. Grounding service delivery in implementation science enables WRAIR to leverage capabilities and partnerships, and enhance quality and sustainability, while securing the PEPFAR priorities of accelerating access to HIV treatment, focusing prevention for maximum effect, and increasing the impact and cost-effectiveness of every dollar invested. Over time, WRAIR’s PEPFAR program has shortened data collection to inform real-time application of findings to help control the epidemic.

## Supplemental tables

Supplemental materials
