# Supplementary figures and images for: Attaining 95-95-95 through Implementation Science: 15 Years of Insights and Best Practices from the Walter Reed Army Institute of Research’s Implementation of the U.S. President’s Emergency Plan for AIDS Relief
Source: Am J Trop Med Hyg. 2020 Nov 9;104(1):12–25. doi: 10.4269/ajtmh.20-0541 (PMC7790083; doi:10.4269/ajtmh.20-0541)

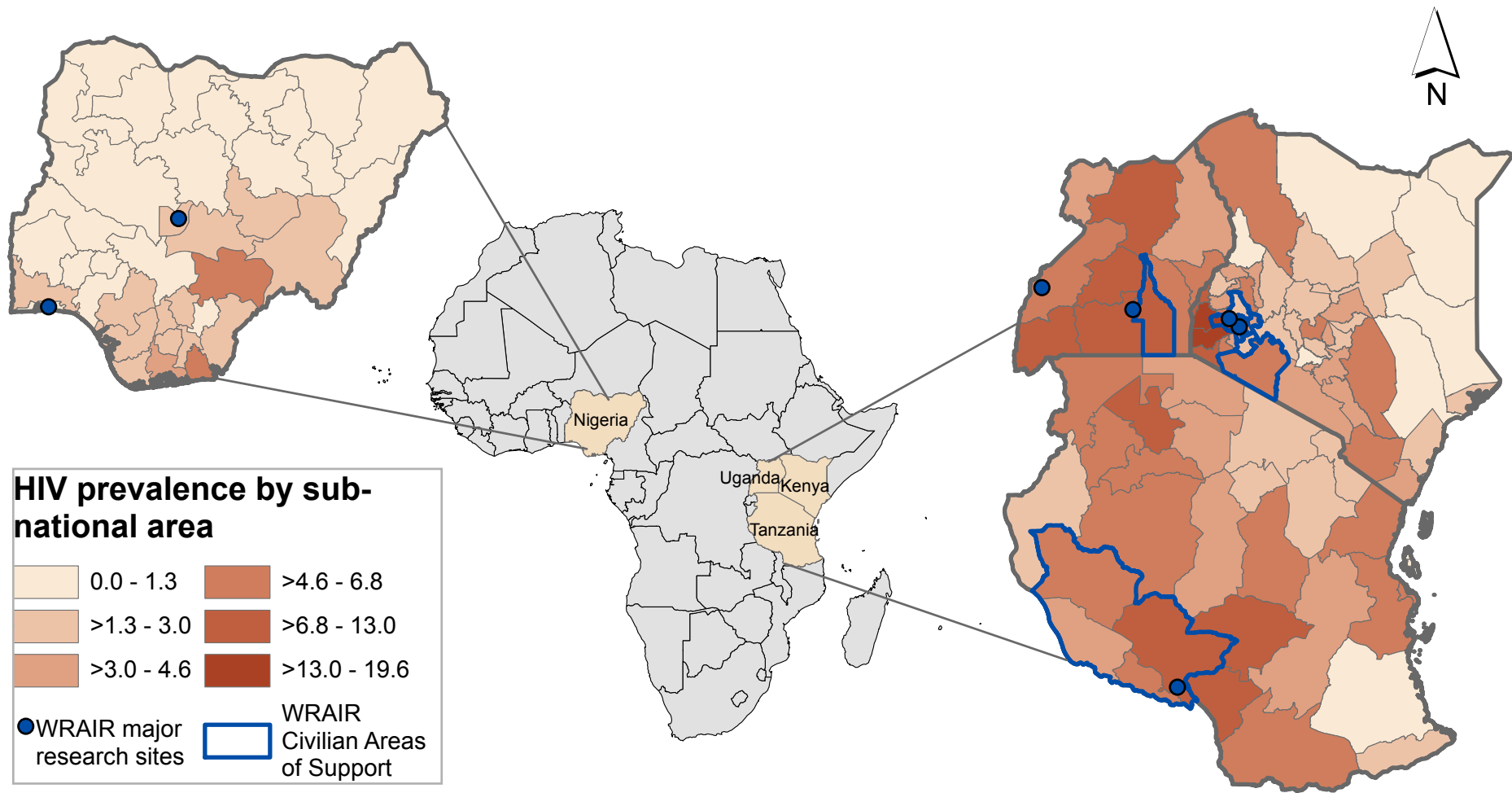

Supplement: Supplementary file 1 [file tpmd200541.SD1.pdf]
