# Supplementary material for: Attaining 95-95-95 through Implementation Science: 15 Years of Insights and Best Practices from the Walter Reed Army Institute of Research’s Implementation of the U.S. President’s Emergency Plan for AIDS Relief
Source: Am J Trop Med Hyg. 2020 Nov 9;104(1):12–25. doi: 10.4269/ajtmh.20-0541 (PMC7790083; doi:10.4269/ajtmh.20-0541)
Supplement: Supplementary file 2 [file tpmd200541.SD2.pdf]

Supplemental Table 1. Walter Reed Army Institute of Research's International HIV Clinical Research Sites in Countries with PEPFAR Programs

Country	Clinical Research Site
Kenya	Kenya Defense Forces, KEMRI, Nairobi
	KEMRI, Nairobi
	KEMRI-WRP, Kericho
	US Military HIV Research Program (MHRP) – Kericho
	Kombewa Clinical Research Centre, Kisumu
	Kombewa District Hospital, Kisumu
	Kapsabet District Hospital, Kapsabet
	Kapkatet District Hospital
	AC Litein District Hospital
	Mtongwe Military Outreach Clinic (MMOC), Kenya Navy Base, Mtongwe, Mombasa
	Nandi Hills District Hospital
	Tenwek Mission Hospital
	Moi University-AMPATH (Academic Model for Prevention and Treatment of HIV), AMPATH Centre at Moi Teaching & Referral Hospital, Eldoret
Tanzania	US Military HIV Research Program (MHRP) HQ, Dar es Salaam
	Mbeya Regional Referral Hospital, Mbeya
	Mbeya Zonal Referral Hospital, Mbeya (includes Baylor)
	National Institute of Medical Research, Mbeya Medical Research Center (NIMR-MMRC), Mbeya
Uganda	Makerere University Walter Reed Project (MUWRP), Kampala
	Fort Portal Regional Referral Hospital, For Portal
	Makerere University Medical School, Kampala
	Kayunga District Hospital
Nigeria	Defense Research Laboratory (DRL), Mogadishu Military Base, Abuja
	Nigerian Clinical Research Center (CRC), Abuja
	45 Nigerian Airforce Hospital, Nigerian Airforce Base, Makurdi
	University of Ibadan, Ibadan
	68 Nigerian Army Reference Hospital, Lagos
	General Hospital Ikorodu, Ikorodu

	Nigerian Air Force Hospital,Sam Ethnam Air Force Base, Lagos
	Community Health Center (CHC), Lagos
	Redeember's University, Ede
	Ikorodu General Hospital, Ikorodu
	Irrua Specialist Teaching Hospital, Irrua

Supplemental Table 2. Published Manuscripts

Date Published	MHRP #/ Clinical Trials #/ ACTG#	Title	Primary Author (Last, First)	Journal
August 26, 2020	RV368*	HIV status disclosure by Nigerian men who have sex with men and transgender women living with HIV	Tiamiyu, Abdulwasiu	BMC Public Health
August 12, 2020	RV329	Pre-Treatment and Acquired Antiretroviral Drug Resistance among Persons Living with HIV in Four African Countries	Crowell, Trevor	Clinical Infectious Diseases
June 21, 2020	RV528	Sustaining tuberculosis preventive therapy scale-up through direct supportive supervision	Meribe, Stanley	Public Health Action
May 31, 2020	RV368*	Multiple HPV infections among men who have sex with men engaged in anal cancer screening in Abuja, Nigeria.	Nowak, Rebecca	Papillomavirus Research
May 8, 2020	RV329	Predictors and Barriers to Condom Use in the African Cohort Study	Analogbei, Tope	AIDS Patient Care and STDs
March 21, 2020	RV555	Taking TB Preventive Therapy implementation to a national Scale: The Nigeria PEPFAR program experience	Bethrand, Odume	Public Health Action
March 3, 2020	RV368*	Transmitted, Pre-Treatment, and Acquired Antiretroviral Drug Resistance among Men who have Sex with Men and Transgender Women Living with HIV in Nigeria	Crowell, Trevor	Antiviral Therapy
March 2, 2020	RV368*	Seizing Opportunities for Intervention: Changing HIV Knowledge among Men who have Sex with Men and Transgender Women Attending Trusted Community Centers in Nigeria	Jones, Milissa	PLoS One
March 1, 2020	RV368*	Anorectal and Urogenital *Mycoplasma Genitalium* in Nigerian Men who have Sex with Men and Transgender Women: Prevalence, Incidence, and Association with HIV	Crowell, Trevor	Sexually Transmitted Diseases
February 26, 2020	RV329	Monocyte Activation, HIV and Cognitive Performance in East Africa	Muñoz-Nevárez, Arnoldo	Journal of Neurovirology
February 17, 2020	RV329	Decreasing time to antiretroviral therapy initiation after HIV diagnosis in a clinic -bas observational cohort study in four African countries	Esber, Allahna	Journal of the International AIDS Society
February 5, 2020	RV368*	Satisfaction with High-resolution Anoscopy for Anal Cancer Screening among Men who have Sex with Men: a Cross-sectional Survey in Abuja, Nigeria	Nowak, Rebecca	BMC Cancer
February 1, 2020	RV368*	Genetic Clustering Analysis for HIV Infection among MSM in Nigeria: Implications for Intervention	Li, Yuruo	AIDS
February 1, 2020	RV329	Frequency and Predictors of HIV-related Cognitive Impairment in East Africa: The Africa Cohort Study (AFRICOS)	Milanini, Benedetta	Journal of Acquired Immune Deficiency Syndromes
October 15, 2019	RV465	A Tool for Estimating Antiretroviral Medication Coverage for HIV-Infected Women During Pregnancy (PMTCT-ACT)	Larson, Bruce	Global Health Research and Policy

September 16, 2019	RV494*	Simplified clinical algorithm for identifying patients eligible for same-day HIV treatment initiation (SLATE): Results from an individually randomized trial in South Africa and Kenya	Rosen, Sydney	PLoS One
September 5, 2019	RV246/ NCT00286767*	Prospective international study of incidence and predictors of immune reconstitution inflammatory syndrome and death in people with HIV and severe lymphopenia	Sereti, Irini	Clinical Infectious Diseases
September 4, 2019	RV516*	Association of the US President's Emergency Plan for AIDS Relief's Funding with Prevention of Mother-to-Child Transmission of HIV in Kenya	Barnhard, Dale	JAMA Network Open
September, 2019	RV494*	Who is seeking antiretroviral treatment for HIV now? Characteristics of patients presenting in Kenya and South Africa in 2017-2018	Brennan, Alana	Journal of Acquired Immune Deficiency Syndrome
August 16, 2019	RV329	Persistent Low-level Viremia Predicts Subsequent Virologic Failure: Is It Time to Change the Third 90?	Esber, Allahna	Clinical Infectious Diseases
August 15, 2019	RV368*	New Subtype B Containing HIV-1 Circulating Recombinant of sub-Saharan Africa Origin in Nigerian Men who have Sex with Men	Billings, Erik	Journal of Acquired Immune Deficiency Syndromes
August 1, 2019	RV329	Noninfectious Comorbidity in the African Cohort Study	Ake, Julie	Clinical Infectious Diseases
July 19, 2019	RV368*	Implementation and early outcomes from an anal cancer screen and treat program at an HIV clinic providing care to Nigerian men who have sex with men	Nowak, Rebecca	Journal of Global Oncology
May 31, 2019	RV329	East African HIV care: depression and HIV outcomes	Meffert, Susan	Global Mental Health
April 1, 2019	RV368*	Individual and Sexual Network Predictors of HIV Incidence among Men who have Sex with Men in Nigeria	Nowak, Rebecca	Journal of Acquired Immune Deficiency Syndromes
March 5, 2019	RV368*	Anal Microbial Patterns and Oncogenic Human Papillomavirus in a Pilot Study of Nigerian Men Who Have Sex with Men at Risk for or Living with HIV	Nowak, Rebecca	AIDS Research and Human Retroviruses
March, 2019	RV368*	Time to change the paradigm: limited condom and lubricant use among Nigerian men who have sex with men and transgender women despite availability and counseling	Crowell, Trevor	Annals of Epidemiology
March 14, 2019	RV367/ NCT01404312/ A5279*	One Month of Rifapentine plus Isoniazid to Prevent HIV-Related Tuberculosis	Swindells, Susan	The New England Journal of Medicine
February 5, 2019	RV329	HIV virologic failure and its predictors among HIV-infected adults on antiretroviral therapy in the African Cohort Study	Kiweewa, Francis	PLoS One
January 7, 2019	RV329	Biomarkers of Inflammation Correlate with Clinical Scoring Indices in HIV-Infected Kenyans	Letizia, Andrew	Journal of Infectious Diseases
December, 2018	RV368*	The Synergistic Impact of Sexual Stigma and Psychosocial Well-Being on HIV Testing: A Mixed-Methods Study Among Nigerian Men who have Sex with Men	Rodriguez-Hart, Christina	AIDS and Behavior

December, 2018	RV368*	Asymptomatic Lymphogranuloma Venereum among Nigerian Men who have Sex with Men	Crowell, Trevor	Sexually Transmitted Infections
November 13, 2018	RV329	Limitations of the International HIV Dementia Scale in the current era	Milanini, Benedetta	AIDS
November, 2018	RV368*	Molecular screening for Neisseria gonorrhoeae antimicrobial resistance markers in Nigerian men who have sex with men and transgender women.	Hardick, Justin	International Journal of STD & AIDS
October 30, 2018	RV465	The Enhanced Mentor Mother ProgrAm (EMMA) for the prevention of mother-to-child transmission of HIV in Kenya: study protocol for a site-randomized controlled trial	Larson, Bruce	Trials
September 1, 2018	RV368*	Individual and Network Factors Associated with HIV Care Continuum Outcomes Among Nigerian MSM Accessing Health Care Services.	Ramadhani, Habib	Journal of Acquired Immune Deficiency Syndromes
August 10, 2018	RV342	Incremental treatment costs for HIV-infected women initiating antiretroviral therapy during pregnancy: a 24-month micro-costing cohort study for a maternal and child health clinic in Kenya	Larson, Bruce	PLoS One
May, 2018	RV368*	Sexual Stigma Patterns among Nigerian Men who Have Sex with Men and Their Link to HIV and Sexually Transmitted Infection Prevalence	Rodriguez-Hart, Cristina	AIDS and Behavior
November, 2017	RV368*	Online Sex-Seeking Among Men who have Sex with Men in Nigeria: Implications for Online Intervention	Stahlman, Shauna	AIDS and Behavior
November 13, 2017	RV368*	Pathways from Sexual Stigma to Incident HIV and Sexually Transmitted Infections among Nigerian MSM	Rodriguez-Hart, Cristina	AIDS
August, 2017	RV368*	Sexual Partner Characteristics and Incident Rectal *Neisseria gonorrhoeae* and *Chlamydia trachomatis* Infections among Gay Men and Other Men who have Sex with Men (MSM): a Prospective Cohort in Abuja and Lagos, Nigeria	Ramadhani, Habib	Sexually Transmitted Infections
July 28, 2017	RV368*	Phylodynamic analysis to inform prevention efforts in mixed HIV epidemics	Volz, Erik	Virus Evolution
June 13, 2017	RV368*	Genotyping of high-risk anal human papillomavirus (HPV): ion torrent-next generation sequencing vs. linear array	Nowak, Rebecca	Virology Journal
April 26, 2017	RV368*	Measuring Sexual Behavior Stigma to Inform Effective HIV Prevention and Treatment Programs for Key Populations	Stahlman, Shauna	JMIR Public Health and Surveillance
April 20, 2017	RV368*	Stigma, access to healthcare, and HIV risks among men who sell sex to men in Nigeria	Crowell, Trevor	Journal of the International AIDS Society
March 27, 2017	RV368*	Rectal Microbiota among HIV-uninfected, Untreated HIV, and Treated HIV-infected in Nigeria	Nowak, Rebecca	AIDS
January 4, 2017	RV257	Excellent retention, virologic and clinical outcomes after transitioning from an antiretroviral treatment clinical trial to locally-provided care and treatment in Africa	Sawe, Fredrick	International Journal of Clinical Trials

December 7, 2016	RV368*	High prevalence of HIV, chlamydia and gonorrhea among men who have sex with men and transgender women attending trusted community centres in Abuja and Lagos, Nigeria	Keshinro, Babajide	Journal of the International AIDS Society
November, 2016	RV368*	Serosorting and Sexual Risk for HIV Infection at the Ego-Alter Dyadic Level: An Egocentric Sexual Network Study Among MSM in Nigeria.	Rodriguez-Hart, Cristina	AIDS and Behavior
July 26, 2016	RV368*	The Prevalence of Sexual Behavior Stigma Affecting Gay Men and Other Men Who Have Sex with Men Across Sub-Saharan Africa and in the United States	Stahlman, Shauna	JMIR Public Health and Surveillance
April 1, 2016	RV368*	Prevalence of Anal High-Risk Human Papillomavirus Infections Among HIV-Positive and HIV-Negative Men Who Have Sex with Men in Nigeria	Nowak, Rebecca	International Journal of STD & AIDS
July, 2015	RV368*	The immediate effect of the Same-Sex Marriage Prohibition Act on stigma, discrimination, and engagement on HIV prevention and treatment services in men who have sex with men in Nigeria: analysis of prospective data from the TRUST cohort.	Schwartz, Sheree	The Lancet HIV
March 16, 2015	RV346	How Much Does It Cost to Improve Access to Voluntary Medical Male Circumcision among High-Risk, Low-Income Communities in Uganda?	Larson, Bruce	PLoS One
March 1, 2015	RV368*	Uptake of treatment as prevention for HIV and continuum of care among HIV-positive men who have sex with men in Nigeria	Charurat, Man	Journal of Acquired Immune Deficiency Syndromes
February 23, 2015	RV257	Kericho CLinic-Based ART Diagnostic Evaluation (CLADE): Design, Accrual, and Baseline Characteristics of a Randomized Controlled Trial Conducted in Predominately Rural, District-Level, HIV Clinics of Kenya	Sawe, Fredrick	PLoS One
August, 2014	RV288	Short Communication: East Meets West: A Description of HIV-1 Drug Resistance Mutation Patterns of Patients Failing First Line Therapy in PEPFAR Clinics from Uganda and Nigeria	Crawford, Keith	AIDS Research and Human Retroviruses
August, 2014	RV288	Evaluation of treatment outcomes for patients on first-line regimens in US President's Emergency Plan for AIDS Relief (PEPFAR) clinics in Uganda: predictors of virological and immunological response from RV288 analyses	Crawford, Keith	HIV Medicine
January 28, 2014	RV288	Occurrence of etravirine/rilpivirine-specific resistance mutations selected by efavirenz and nevirapine in Kenyan patients with non-B HIV-1 subtypes failing antiretroviral therapy	Crawford, Keith	AIDS
November 28, 2011	RV563	Successes and Challenges in an Integrated Tuberculosis/HIV Clinic in a Rural, Resource-Limited Setting: Experiences from Kericho, Kenya	Shaffer, Douglas	AIDS Research and Treatment
October 14, 2010	RV186/ NCT00089505/ A5208*	Antiretroviral Therapies in Women after Single-Dose Nevirapine Exposure	Lockman, Shahin	The New England Journal of Medicine

*Select WRAIR Implementation Science Studies with outside funding that leverage PEPFAR sites

Supplemental Table 3. Abstracts and Presentations

Conference/ Meeting Name and Year	Study ID	Presentation Type	Title	Primary Author (Last, First)
International Union Against Sexually Transmitted Infections	RV432	Oral	HIV Risk Taking Behaviors among Fishing Communities on Lake Victoria in Uganda	Akom, Eniko
INTEREST 2020	RV432	Poster	Factors Associated with High HIV Prevalence among Fisherfolk on Lake Victoria, Uganda	Mwesigwa, Betty
INTEREST 2020	RV543	Poster	Predictors of HIV Infection Among HIV Exposed Babies Infants Receiving PMTCT services in Nigerian Military Hospitals	Ahmed, Yusuf
INTEREST 2020	RV476	Poster	Adherence among Adolescent Girls and Young Women Sex Workers on HIV Oral Pre-Exposure Prophylaxis in Mukono District, Uganda	Mirembe, Grace
INTEREST 2020	RV432	Poster	Factors Associated with Condom Use among Fishing Communities on Koome and Buvuma Islands, Uganda	Akom, Eniko
AIDS 2020	RV543	Poster	Social and Dating Online Platforms as an Opportunity to Reach Nigerian MSM with HIV Services. The Experience of the Lagos Community Health Centre	Okonkwo, Dooshima
AIDS 2020	RV476	Poster	Side effects, life transitions, and disclosure: Reasons for oral pre-exposure prophylaxis (PrEP) discontinuation among young women engaged in sex work in Uganda.	Mathur, Sanyukta
AIDS 2020	RV465	Poster	Antiretroviral medication coverage along the PMTCT cascade of care in the era of treatment for all in Kenya: results from a pragmatic, cluster randomized trial (the EMMA study)	Larson, Bruce
AIDS 2020	RV329	Poster	Routine HIV Clinic appointment adherence in the African Cohort Study	Dear, Nicole
AIDS 2020	RV329	Oral	Weight Gain and Hyperglycemia during the Dolutegravir Transition in Africa	Ake, Julie
AIDS 2020	RV449	Poster	Finding the Missing People Living with HIV and AIDS in the Southern Highland Zone, Tanzania: Strategic U "the use of a National HIV Testing Services (HTS) Eligibility Screening Tool	Ngwalle, Abel
AIDS 2020	RV449	Poster	Improving linkage to antiretroviral treatment using a peer attachment model in the selected facilities in the Southern Highland Zone, Tanzania	Ngwalle, Abel
International Conference on HIV Treatment and Prevention Adherence 2020	RV559	Poster	Improving Viral Load Suppression among Youth in the Nigeria Ministry of Defense HIV Program	Lawal, Ismail
International Social and Behavior Change Communication Summit 2020	RV476	Poster	My Eighteen-Year Old Girl Will Start Going in for Men, Knowing She Can't Contract HIV": Ugandan Health Care Workers' knowledge and Attitudes on PrEP – a Mixed Methods Study.	Akom, Eniko
International Social and Behavior Change Communication Summit 2020	RV563	Poster	Give them economic empowerment, keep them HIV free!	Goretty, Sarah
World Ophthalmology Congress 2019	RV329	Poster	Ophthalmic Disease in the AFRICOS HIV-Positive Cohort	Grant, Justin

SHARe Symposium 2019	RV543	Oral	Making sense of Health program data	Meribe, Stanley
SHARe Symposium 2019	RV543	Oral	Contact Elicitation Ratio in Index Testing Services Among PLHIV Accessing Services in Military Hospitals in Nigeria	Agweye, Angela
SHARe Symposium 2019	RV543	Oral	Improving Site Level Performance through Enhanced Site Management and Continuous Quality Improvement: Experience from the Nigeria Military HIV Program	Uzim, Justus
SHARe Symposium 2019	RV543	Poster	Improving Data Demand and Use in the Nigerian Military HIV Program	Adebayo-Abikoye, Esther
American Society of Tropical Medicine and Hygiene Annual Meeting 2019	RV543	Poster	Building Global Health Security Workforce and Research Infrastructure through North-South Collaboration: The U.S-Nigeria Military Experience	Chittenden, Laura
AFREHealth Symposium 2019	RV557	Oral	Towards GeneXpert system optimization and improving tuberculosis (TB) case identification: The Nigerian military program experience	Meribe, Stanley
Fast Track Cities 2019	RV543	Poster	Improving Viral Load Suppression in Nigerian Adolescents 10-24 Years through an Adolescent-friendly Service Package	Lawal, Ismail
AIDS 2019	RV329	Poster	Characterizing Metabolic Syndrome in HIV-infected Individuals in Sub-Saharan Africa: Does the definition matter?	Esber, Allahna
AIDS 2019	RV329	Poster	Temporal trends in HIV-related self-reported stigma in the African Cohort Study, 2013-2018	Esber, Allahna
AIDS 2019	RV 415,449,543,563	Poster	A Look at Children Living with HIV and on Treatment in PEPFAR-Supported Programs: 2017 – 2018	PEPFAR Interagency
Union World Conference on Lung Health 2019	RV557	Poster	Direct Supportive Supervision results in improved isoniazid preventative therapy implementation in the Nigerian Military HIV program	Meribe, Stanley
INTEREST 2019	RV329	Poster	Trend of Virological Response with Second Line ART Switch in an African Cohort Population	Agbaim, Uzoamaka
INTEREST 2019	RV449	Poster	The Effectiveness of Option B+ among Children born to Women Living with HIV in Mbeya and Songwe Regions	Philemon, Edson
INTEREST 2019	RV449	Poster	Experience on the Implementation of Assisted Partner Notification Services for Mbeya and Songwe Regions in July, 2018	Mgaya, Jimson
INTEREST 2019	RV449	Poster	One-year experience of Methadone Clinic at Mbeya Zonal Referral Hospital	Kiwera, Rehema
East African Health and Scientific Conference 2019	RV329	Poster	Satisfaction with ART Care and its Association with Viral Suppression in Africa	Somi, Nancy
East African Health and Scientific Conference 2019	RV329	Poster	HIV Controllers in Four African Countries	Lwilla, Anange

International Workshop on HIV and Hepatitis Observational Databases 2019	RV329	Poster	Temporal trends in HIV-related self-reported stigma in the African Cohort Study, 2013-2018	Esber, Allahna
Conference on Retroviruses and Opportunistic Infections 2019	RV329	Poster	Antiretroviral Therapy Regimen Durability in a Large African Cohort	Polyak, Christina
Conference on Retroviruses and Opportunistic Infections 2019	RV329	Poster	Trend of Viral Suppression with Second Line ART Switch in an African Cohort Population	Agbaim, Uzoamaka
Conference on Retroviruses and Opportunistic Infections 2019	RV329	Poster	HIV Controllers in Four African Countries	Lwilla, Anange
Conference on Retroviruses and Opportunistic Infections 2019	RV329	Poster	HIV Infected Adults Perceived Satisfaction with Care and its Association with Viral Suppression in the African Cohort Study.	Somi, Nancy
SHARe Symposium 2018	RV528	Poster	"WHO Test and Start" Strategy and Programmatic Implications for TB-HIV in Nigeria: An evaluation of treatment program in 27 Nigerian Military Hospitals	Meribe, Stanley
African Society for Laboratory Medicine 2018	RV543	Poster	Strategies for Assuring the Quality of HIV Testing in Nigerian Military Facilities	Suleiman, Aminu
African Society for Laboratory Medicine 2018	RV449	Poster	Using an MHealth intervention to link health facilities and testing laboratories in improving the uptake of viral load testing in southern Tanzania	Khamadi, Samoel
African Society for Laboratory Medicine 2018	RV528	Poster	"WHO Test and Start" Strategy and Programmatic Implications for TB-HIV in Nigeria: An evaluation of treatment program in 27 Nigerian Military Hospitals	Meribe, Stanley
African Society for Laboratory Medicine 2018	RV563	Poster	Supporting national viral load (VL) and early infant diagnosis (EID) scale-up: Experience from Clinical Research Centre (CRC) Laboratory in Kericho, Kenya	Kasembeli, Alex
African Society for Laboratory Medicine 2018	RV449	Poster	A review of Pre-Analytical Errors at a Tertiary Health Facility in the Southern Highlands of Tanzania	Somi, Nancy
African Society for Laboratory Medicine 2018	RV449	Poster	Expanding HIV Viral Load Testing in Tanzania: Evaluation of the VERSANT HIV-1 RNA Assay	Nkika, Busagwe Zablon
Muhimbili University of Health and Allied Sciences, Scientific Conference 2018	RV329	Poster	Depression and its Associated Factors among AFRICOS Study Participants at Mbeya Zonal Referral Hospital	Lwilla, Anange
Muhimbili University of Health and Allied Sciences, Scientific Conference 2018	RV399	Poster	Factors associated with HIV infection among children born to mothers on the prevention of mother to child transmission (PMTCT) program in Mbeya and Songwe Regions, 2016	George, Fredy

Muhimbili University of Health and Allied Sciences, Scientific Conference 2018	RV399	Poster	Does mobile phone ownership improve access to EID services in the Southern Highlands of Tanzania?	Kafwimi, Erika
The National AIDS and STI's Control Programme Conference 2018	RV329	Poster	Cervical cancer screening program in South Rift Valley, Kisumu West, Kenya: Findings of the screening program	Kimetto, Jane
The National AIDS and STI's Control Programme Conference 2018	RV329	Poster	Weight Status and associated factors among HIV-infected Adults in the African Cohort Study	Langat, Rither
HIV Research for Prevention 2018	RV329	Poster	Association between Discordance status, ART use and viral suppression in the African Cohort Study (AFRICOS)	Reed, Domonique
American Society of Tropical Medicine and Hygiene Annual Meeting 2018	RV329	Poster	Global Health Engagement via the African Cohort Study: Quantifying the Prevalence and Incidence of HIV-Associated Co-Infections in Sub-Saharan Africa	Polyak, Christina
Union World Conference on Lung Health 2018	RV555	Oral	Taking TB Preventive Therapy implementation to a national Scale: The Nigeria PEPFAR program experience	Meribe, Stanley
Quebec Population Health Research Network 2018	RV 415,449,543,563	Poster	Mobile technology for HIV programs and research: successes and ethical pitfalls	Akom, Eniko
INTEREST 2018	RV329	Poster	Predictors of and Barriers to Condom Use in the AFRICAN Cohort Study	Analogbei, Tope
INTEREST 2018	RV329	Poster	Weight Status and associated factors among HIV-infected Adults in the African Cohort Study	Langat, Rither
INTEREST 2018	RV432	Poster	Human Immunodeficiency Virus, Syphilis and Hepatitis B Virus Infections in the Fishing Communities of Koome and Buvuma Islands in Uganda in the Era of Universal Antiretroviral Therapy	Kiweewa, Francis
AIDS 2018	RV329	Poster	Immune Activation Parameters are differentially expressed across four countries in sub-Saharan Africa and are associated with comorbidities in HIV+ and HIV- individuals	Streeck, Hendrik
AIDS 2018	RV329	Poster	Association between Discordance status and ARV use in the African Cohort Study	Reed, Domonique
AIDS 2018	RV329	Poster	Gender Based Differences in Clinical Characteristics and Social Barriers to HIV Care Among HIV-Infected Participants in Four African Countries	Ganesan, Kavitha
AIDS 2018	RV329	Poster	Prevalence of and Factors Associated with Anemia among HIV-infected Adults in a Multi-Site African Cohort	Parikh, Ajay
AIDS 2018	RV329	Poster	Assessing the clinical significance of persistent low level viremia on subsequent virologic failure	Esber, Allahna

AIDS 2018	RV516	Oral	The impact of PEPFAR PMTCT funding on reduced infant mortality and improved ANC care in Kenya: A quasi-experimental evaluation	Barnhard, D.A.
Society for Epidemiologic Research 2018	RV516	Poster	The impact of PEPFAR PMTCT funding on reduced infant mortality and improved ANC care in Kenya: A quasi-experimental evaluation	Barnhard, D.A.
International Workshop on HIV and Hepatitis Observational Databases 2018	RV329	Poster	Assessing the clinical significance of persistent low level viremia on subsequent virologic failure	Esber, Allahna
International Social and Behavior Change Communication Summit 2018	NA	Poster	Stigma no more! How a military community managed to overcome stigma against HIV-infected people	Uganden, Dooshima
Conference on Retroviruses and Opportunistic Infections 2018	RV329	Poster	Predictors of Persistent Low Level Viremia and Transient Viral Blips	Esber, Allahna
Conference on Retroviruses and Opportunistic Infections 2018	RV329	Poster	Predictor of HIV-related Cognitive Impairment in East Africa	Milanini, Benedetta
Human Quality Assessment Services 2018	RV 415,449,543,563	Oral	Is Laboratory Medicine A Barrier to Effective Health Care in Africa?	Oundo, Joe
American Society of Tropical Medicine and Hygiene Annual Meeting 2017	RVR329	Oral	Rate of viremia and its predictors among adult HIV-infected adults in the African HIV Cohort Study	Kiweewa, Francis
American Society of Tropical Medicine and Hygiene Annual Meeting 2017	RV329	Oral	Cryptococcal Antigenemia in African Cohort Study	Sing'oei, Valentine
American Society of Tropical Medicine and Hygiene Annual Meeting 2017	RV330	Poster	Rates of Tuberculosis Diagnosis Among an HIV-Positive Cohort in 4 African Countries	Ganesan, Kavitha
American Society of Tropical Medicine and Hygiene Annual Meeting 2017	RV329	Oral	Demographic and Contextual factors associated with HIV Mortality in an African Cohort in East Africa and Nigeria	Kibuuka, Hannah
Ethiopia Laboratory Technical Working Group 2017	NA	Oral	Continual Quality Improvement and Accreditation	Akinwale, E.A.
Conference on Retroviruses and Opportunistic Infections 2017	RV329	Poster	HIV Drug Resistance - A Unique Perspective Across Four African Countries	Danboise, Brooke
Conference on Retroviruses and Opportunistic Infections 2017	RV329	Poster	Limitations of the International HIV Dementia Scale in the Current Era	Milanini, Benedetta
Center for AIDS Research Symposium 2016	RV329	Oral	Treatment Initiation at Higher CD4s: Challenges to HIV Care in Four African Countries	Polyak, Christina
Military Health System Research Symposium 2016	RV329	Poster	Treatment Initiation at Higher CD4s: Challenges to HIV Care in Four African Countries	Polyak, Christina
Military Health System Research Symposium 2016	RV329	Poster	Decreasing Time to ART Initiation after HIV Diagnosis in Sub-Saharan Africa	Crowell, Trevor
International Symposium on Cervical Cancer in Sub-Saharan Africa	RV329	Oral	Cervical Cancer Screening in the African Cohort Study	Ake, Julie

AIDS 2016	RV329	Poster	Treatment Initiation at Higher CD4s: Challenges to HIV Care in Four African Countries	Polyak, Christina
AIDS 2016	RV329	Oral	The African Cohort Study (AFRICOS)	Coakley, Peter
Keystone Symposia on Molecular and Cellular Biology 2015	RV329	Poster	Matrix Metalloproteinases in Treated and Untreated HIV-1 Infected Kenyans	Eller, Michael
Infectious Disease Week 2015	RV329	Poster	Comparison of Inflammatory Biomarkers and Immune Activation with Comorbid Conditions in a Kenyan Population Based upon HIV Status and Treatment	Letizia, Andrew
American Society of Tropical Medicine and Hygiene Annual Meeting 2015	RV329	Poster	Malaria infection (symptomatic and asymptomatic) among HIV clinic clients in the African Cohort Study (AFRICOS)	Cowden, Jessica
International NeuroHIV Cure Consortium - 2015	RV329	Oral	RV329: African Cohort Study (AFRICOS) NP Analyses Progress Report	Valcour, Victor
AIDS 2015	RV329	Oral	The African Cohort Study (AFRICOS) Informing Progress to 90-90-90	Coakley, Peter
UCSF East Africa Collaborative Scientific Symposium 2015	RV329	Oral	Limited Utility of the international HIV dementia scale in East Africa	Langat, Rither
Conference on Retroviruses and Opportunistic Infections 2015	RV329	Poster	Infectious and Noninfectious Multimorbidity Among HIV Clinic Clients in the African Cohort Study	Ake, Julie
Conference on Retroviruses and Opportunistic Infections 2015	RV329	Poster	Predictors of Cognitive Performance Among HIV-Infected Patients in East Africa	Valcour, Victor
Medical and Veterinary Virus Research Symposium 2014	RV329	Oral	African Cohort Study (AFRICOS): baseline Cohort Characteristics from the Tanzania Site	Mizinduko, Mucho
Tanzania Commission for AIDS 2014	RV329	Oral	African Cohort Study (AFRICOS) - Early Enrollment and Cohort Experience	Mizinduko, Mucho
HIV Research for Prevention 2014	RV329	Oral	Informing Prevention Across Programs: Applications of the African Cohort Study (AFRICOS	Ake, Julie
AIDS 2014	RV329	Poster	Cellular and Soluble Immune Activation Markers are Associated with HIV-1 Infection in Rural Uganda	Omalla, Allan
Conference on Retroviruses and Opportunistic Infections 2014	RV329	Poster	Etravirine/Rilpivirine-Specific Mutations Selected by Efavirenz and Nevirapine in Kenyan Patients Failing ART	Crawford, Keith
American Society of Tropical Medicine and Hygiene Annual Meeting 2013	RV329	Poster	The African Cohort Study (AFRICOS): Early enrollment and opportunities for collaboration	Kiweewa, Francis
AIDS 2013	RV329	Poster	Evaluation of Treatment Outcomes on First-Line Regimens in PEPFAR Clinics in Uganda: Predictors of Virologic and Immunologic Response from RV288 Analyses	Crawford, Keith
Conference on Retroviruses and Opportunistic Infections 2013	RV257	Oral	Superiority of Routine Viral Load Monitoring in Rural Kenya: the Kericho Clinic-based ART Diagnostic Evaluation (CLADE) Trial	Sawe, Fredrick
Conference on Retroviruses and Opportunistic Infections 2013	RV257	Poster	Early Hospitalization and Mortality in Patients Starting ART in Rural Kenya: the Kericho Clinic-based ART Diagnostic Evaluation (CLADE) Trial	Obiero, Eunice
